# Supplementary material for: Inhibitors of eIF1A-ribosome interaction unveil uORF-dependent regulation of translation initiation and antitumor and antiviral effects
Source: EMBO J. 2025 May 12;44(13):3853–71. doi: 10.1038/s44318-025-00449-6 (PMC12216609; doi:10.1038/s44318-025-00449-6)
Supplement: Supplementary file 1 — Appendix [file 44318_2025_449_MOESM1_ESM.pdf]

Appendix for

Inhibitors of eIF1A-ribosome interaction unveil uORF-dependent regulation of translation initiation and antitumor and antiviral effects

Table of content

Appendix Figure S1...................................... 1

Appendix Figure S2...................................... 2

Appendix Figure S3...................................... 3

Appendix Figure S4...................................... 4

Appendix Figure S5...................................... 5

Appendix Figure S6...................................... 6

Appendix Figure S7...................................... 7

Appendix Figure S8...................................... 8

Appendix Table S1.. 9

Appendix Table S2...................................... 13

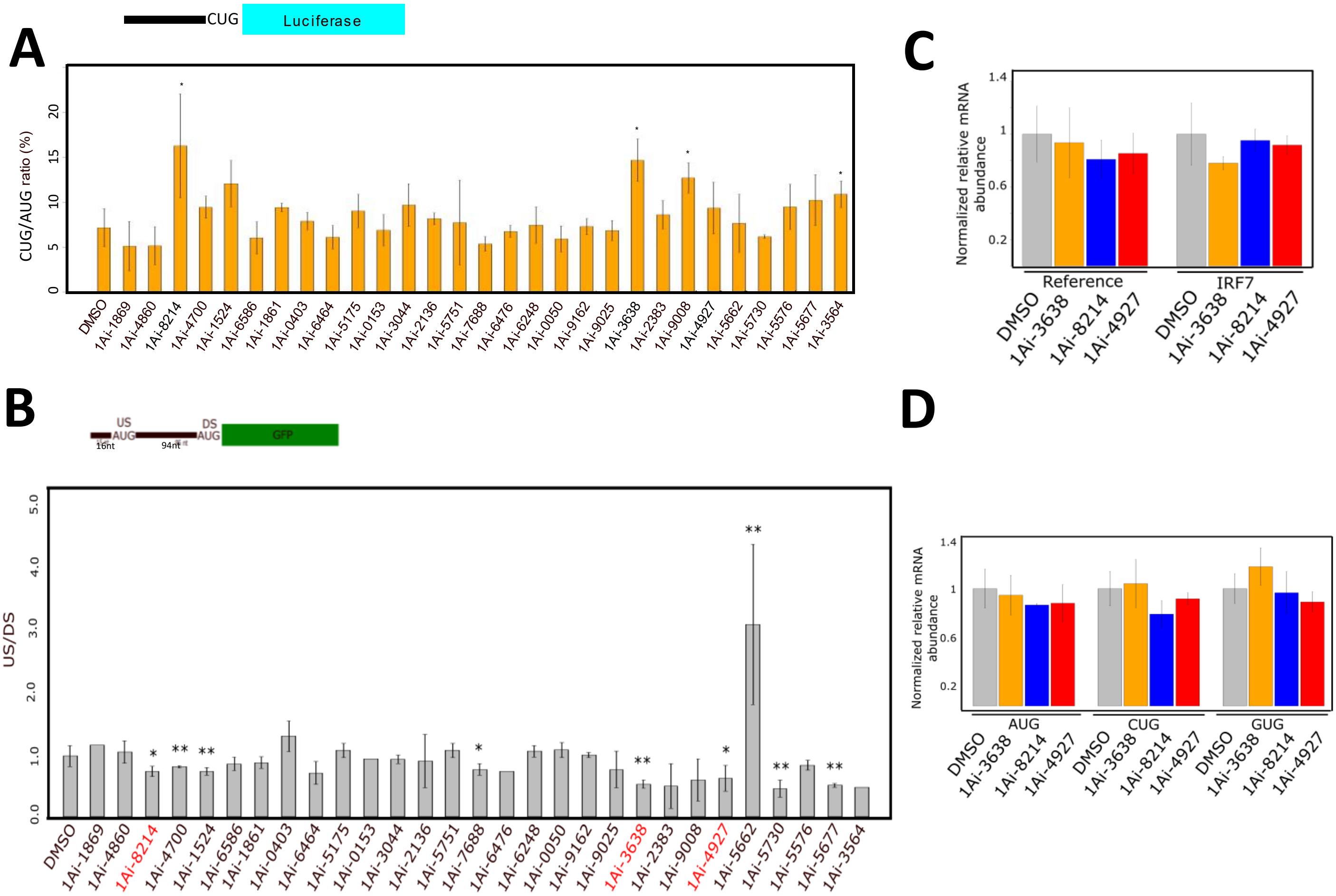

Appendix Figure S1: (A) HEK293T cells were transfected with Luciferase reporter genes with either CUG or AUG as their exclusive starting codon. 6 hours after transfection, cells were exposed to 1Ais, and the luminescence from all constructs was measured after overnight incubation. Bars represent the ratio between luminescence from the AUG starting codon and CUG after normalization with co-transfected GFP; Error bars represent SEM, * denotes $p<0.05$ (T-test comparing to DMSO control), n=≥4 independent biological replicates. (B) 1Ais affect leaky scanning emerging from very short 5'UTR. HEK293T cells were transfected with the GFP reporter gene schematically shown on the top. The first AUG is 15 nt from the 5' end and the downstream AUG is in the same frame. Six hours after transfection, cells were treated with 1Ais. After overnight exposure to 1Ais, GFP expression was analyzed by WB using anti-GFP antibodies. US/DS ratio was determined by densitometry and represents the long protein expression (upstream AUG, US) vs the short protein expression ratio (downstream AUG, DS); Error bars represent SEM, *$p<0.05$, **$p<0.01$, n≥4 independent biological replicates. (C-D) Analysis of reporter genes mRNA levels after 1Ais treatment. HEK293T cells were transfected with Luciferase reporter genes with either IRF7, Reference Firefly, GUG, CUG or AUG. 6 hours after transfection, cells were exposed to 1Ais and 24 hours after transfection mRNA was harvested using Trisol/ethanol precipitation followed by DNase I treatment. mRNA was then reverse transcribed to cDNA and mRNA levels were measured by qPCR using Firefly specific primers. For each sample, we run a –RT sample as a control. Results are presented as mRNA abundance after 1Ais treatment relative to DMSO treatment; Error bars represent SEM, n=3 independent biological replicates.

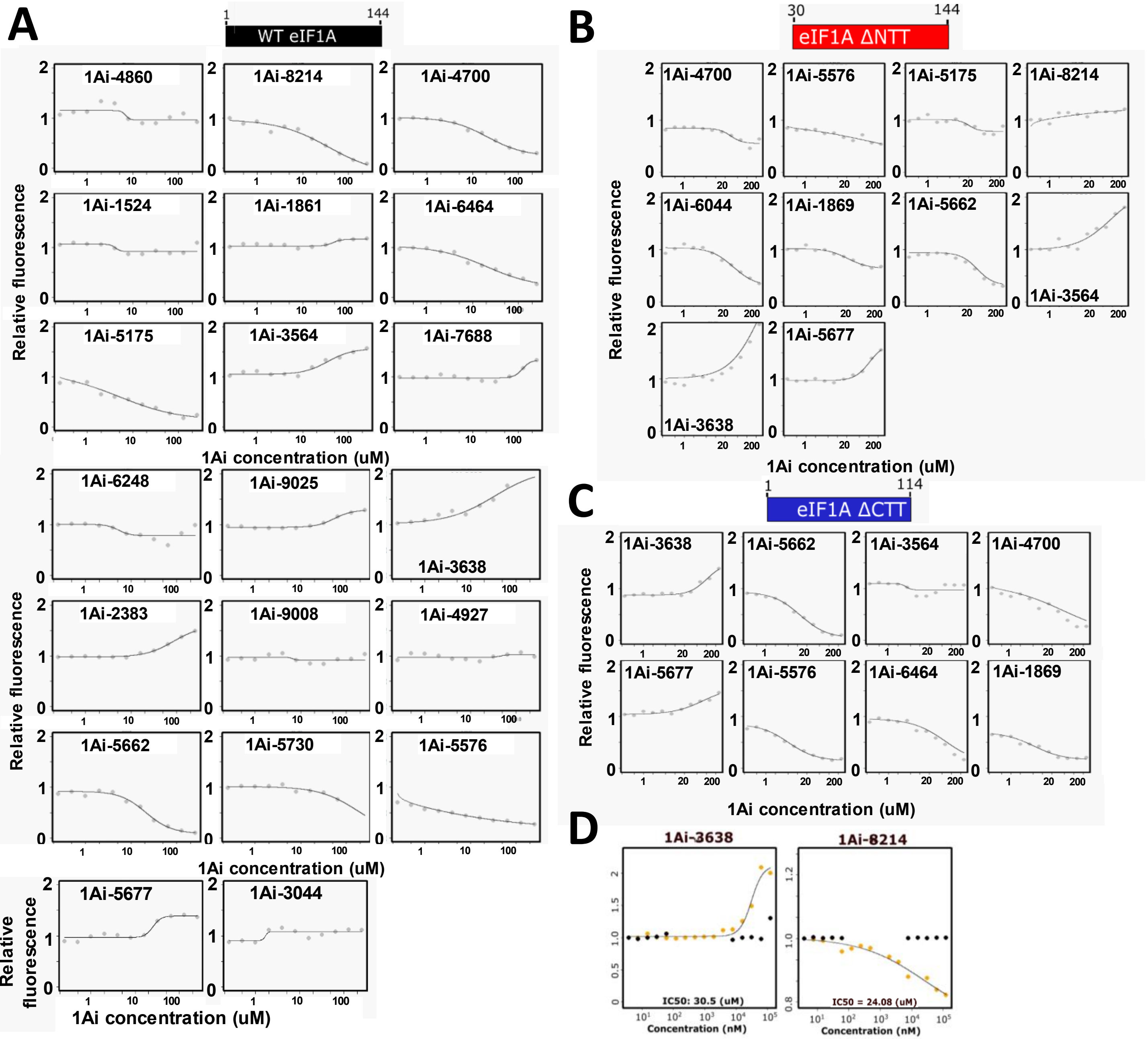

<u>**Appendix Figure S2**</u>: Binding plots of the different 1Ais bound to purified and fluorescently labeled eIF1A. (A) WT eIF1A, (B) eIF1A without NTT, (C) eIF1A without CTT. The WT and mutant eIF1A proteins were expressed as recombinant proteins in bacteria. Purified proteins were incubated with 1Ais with increasing drug concentrations (0.25 µM -250µM). After 10 minutes of incubation, fluorescence intensity was measured (grey dots); n=2. (D) Measurements of changes in fluorescence with MST. eIF1A was purified from a bacterial lysate and labeled with fluorescent dye. Next, eIF1A was incubated with increasing 1Ais concentration, centrifuged, and loaded on MST capillaries. After fluorescence was measured (orange dots), samples were denatured by incubation for 1 hour in 4M Urea. Fluorescence of the highest and lowest five concentrations was measured again (black dots). Curve fitting was done using the ATTbioquest IC50 calculator online tool (35).

A

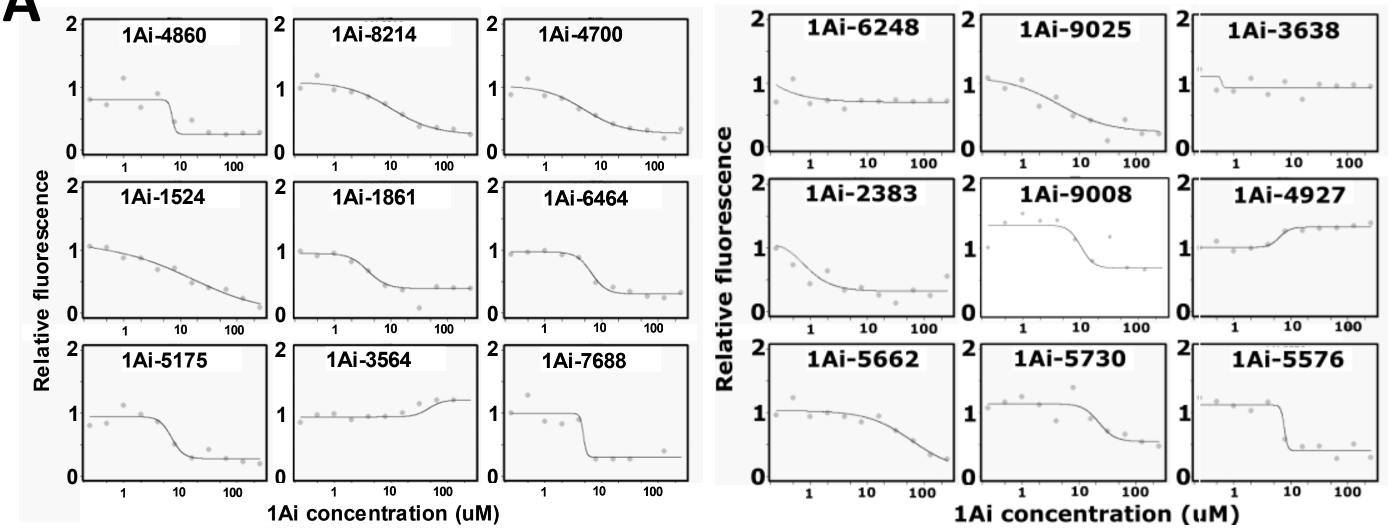

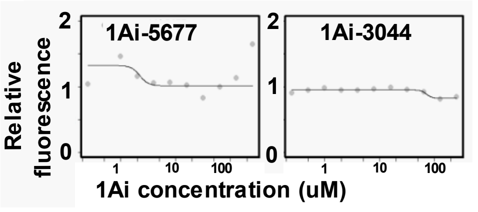

B

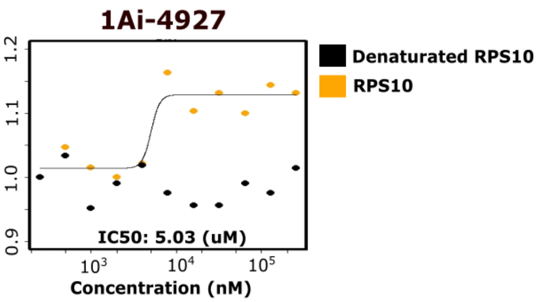

C

	IC50 concentration (μM)				
1Ai	IC50 screen	WT eIF1A	NTT truncation	CTT truncation	RPS10
3638	23	34.1	29.5	84.0	No binding
3564	15	35.3	43.2	No binding	No binding
5677	15	24.8	108.8	79.9	No binding
5662	25	20.9	37.9	12.4	No binding
4700	11	20.9	45.7	30.2	4.9
1869	14	20.4	21.3	5.7	7.7
5576	24	0.9	113.8	5.4	7.4
6464	13	21.2	37.0	35.4	6.4
5175	16	6.0	No binding		6.7
8214	17	37.4	No binding		10.0
4927	14	No binding			6.1
1524	30	No binding			16.8
2383	23	No binding			0.7
7688	20	No binding			4.5
1861	15	No binding			3.7
5730	24	No binding			22.1
9025	17	No binding			4.8
9008	27	No binding			9.9
3044	10	No binding			No binding
6248	28	No binding			No binding
4860	23	No binding			No binding

Appendix Figure S3: (A) Binding plots of the different 1Ais with purified and fluorescently labeled RPS10. RPS10 was expressed as recombinant proteins in bacteria. Purified protein was incubated with 1Ais at increasing drug concentrations (0.25 μM -250μM). After 10 minutes of incubation fluorescence intensity was measured (grey dots). (B) Binding plots of 1Ai-4927 with purified RPS10 (orange) and denatured RPS10 (black) . Experimental procedure for RPS10 fluorescence are the same as figure S3A. After fluorescence measurement samples denatured in 4M Urea before fluorescence was measured again. (C) A table summarizing all IC50 measurements in plots from figures S2A-C and S3A. The binding curve and IC50 were calculated using the Bioquest online tool for IC50 calculations; n≥2.

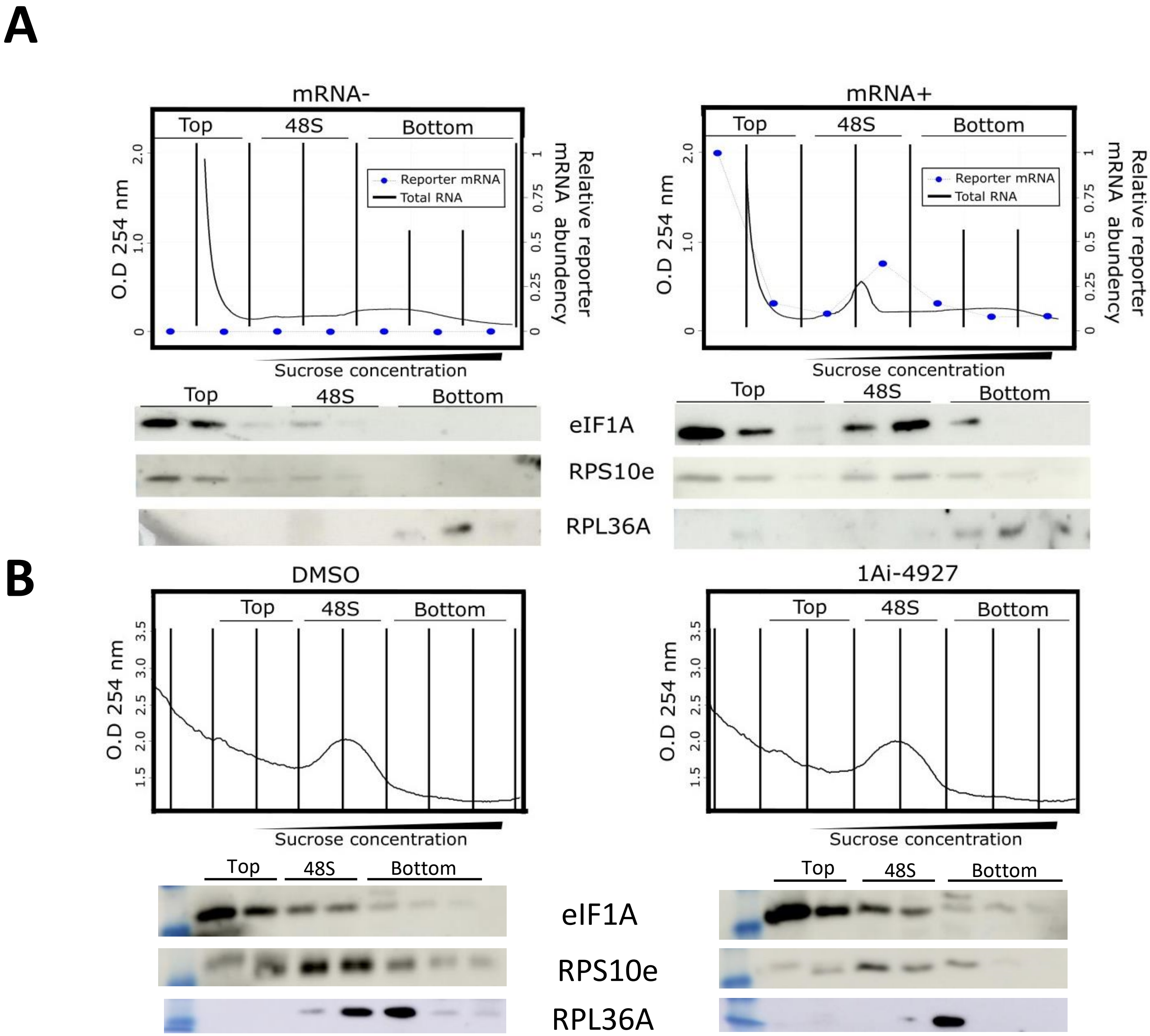

Appendix Figure S4: Biochemical characterization of 1Ais interaction with eIF1A and RPS10 and the 48S complex. (A) A control experiment examining the 48S complexes formation in the presence or absence of an *in-vitro* synthesized luciferase mRNA. Rabbit Reticulocyte lysates (RRL) were pre-incubated with DMSO, GMP-PNP and *in-vitro* synthesized luciferase mRNA (right panel) or water (left panel). The 48S complexes were separated on a sucrose gradient and the O.D at 254 nm was measured (black lines, left Y-axis). Each gradient was separated into top, 48S, and bottom fractions according to the 48S peak location and samples were taken from each fraction for cDNA preparation and WB analysis. Luciferase cDNA from each fraction was quantified using qPCR (blue dots, right Y-axis), while the rest of each fraction was subjected to TCA precipitation followed by WB testing for the presence of the indicated proteins. (B) 48S formation in vitro in the absence or presence of 1Ai-4927. Rabbit Reticulocyte lysates (RRL) were pre-incubated with 1Ai-4927 or DMSO, and then 48S complexes were formed by adding GMP-PNP and in vitro synthesized luciferase mRNA. The 48S complexes were separated on a sucrose gradient and the O.D at 254 nm was measured. Each gradient was separated into top, 48S, and bottom fractions according to the 48S peak location, and samples from each fraction were subjected to TCA precipitation followed by WB testing for the presence of the indicated proteins.

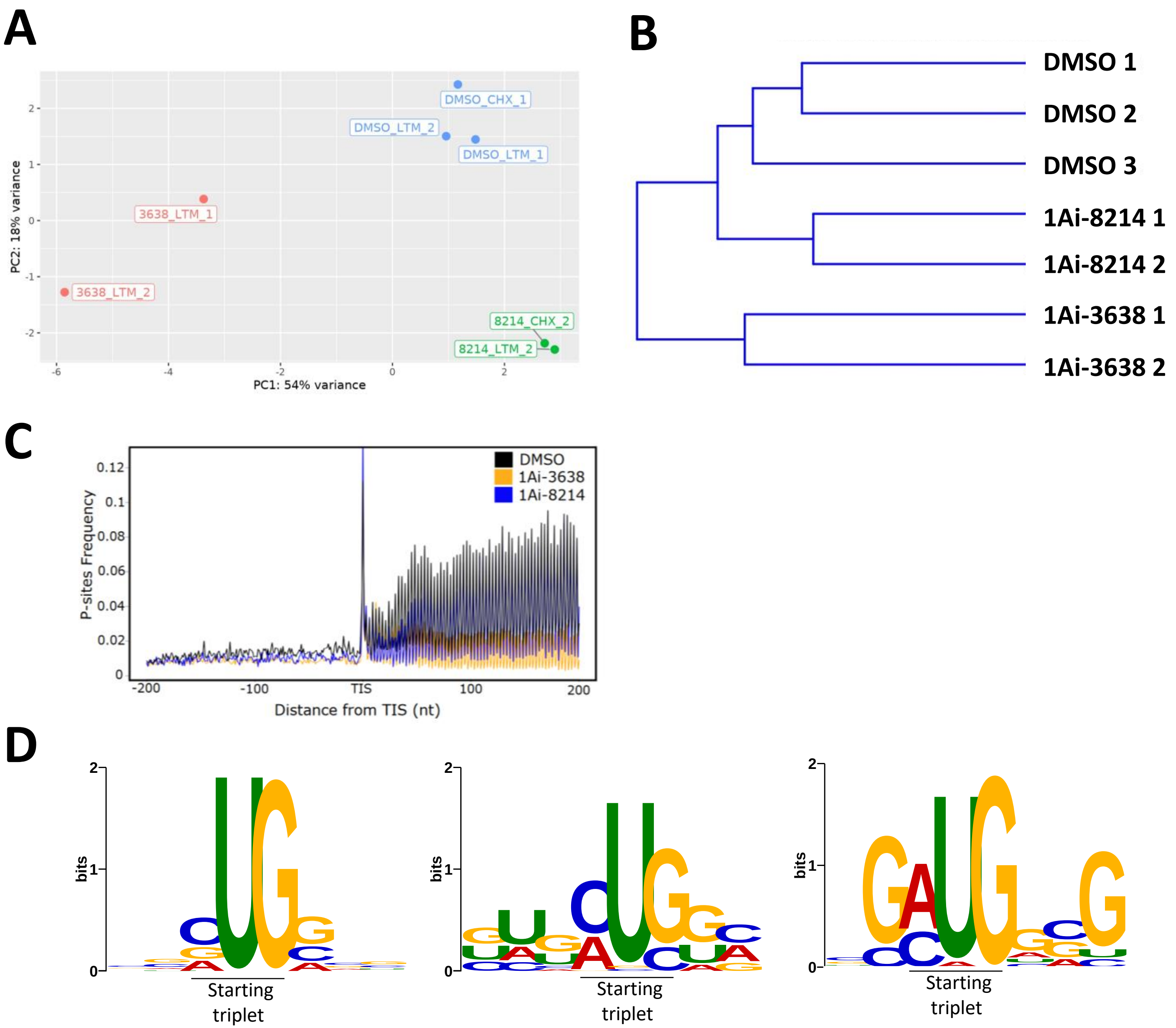

Appendix Figure S5: Ribo-seq analysis. (A) PCA analysis for the samples used for data analysis. (B) Correlation distances between selected samples used for data analysis. (C) Metagene analysis. The p-site in each ribosome footprint was located and the ribosomal occupancy of each area on the mRNA was calculated and normalized to mRNA total counts. Data is normalized to the TIS peak. (D) AUG context of uORFs starting codons. Genes downregulated by 1Ai-3638, 1Ai-8214 and unaffected genes 5'UTR sequence were analyzed for uORFs presence according to previous TIS-seq data (28). Logos present nucleotides flanking the initiation site triplet. All data analysis and clustering were done using Bioconductor package in R.

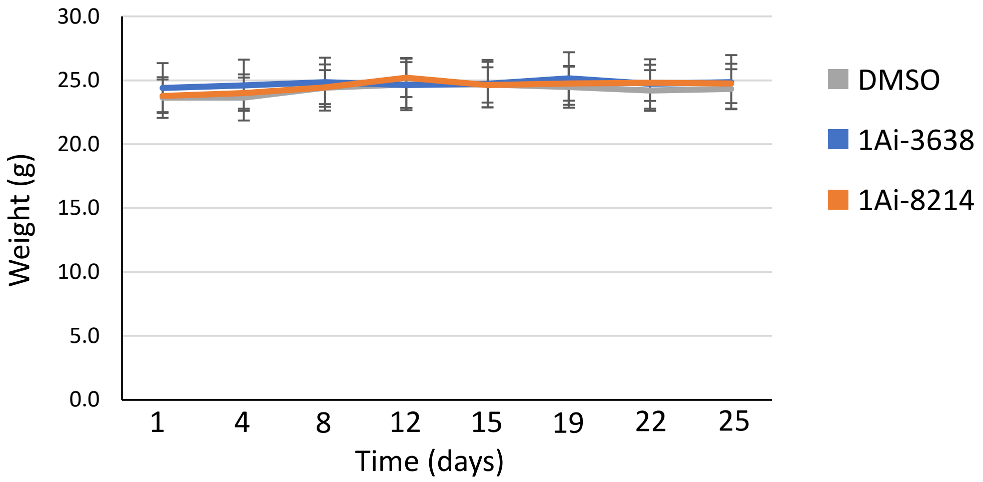

<u>Appendix Figure S6:</u> Weight measurement of mice during 1Ais treatment after OVCAR8 xenografts formation.

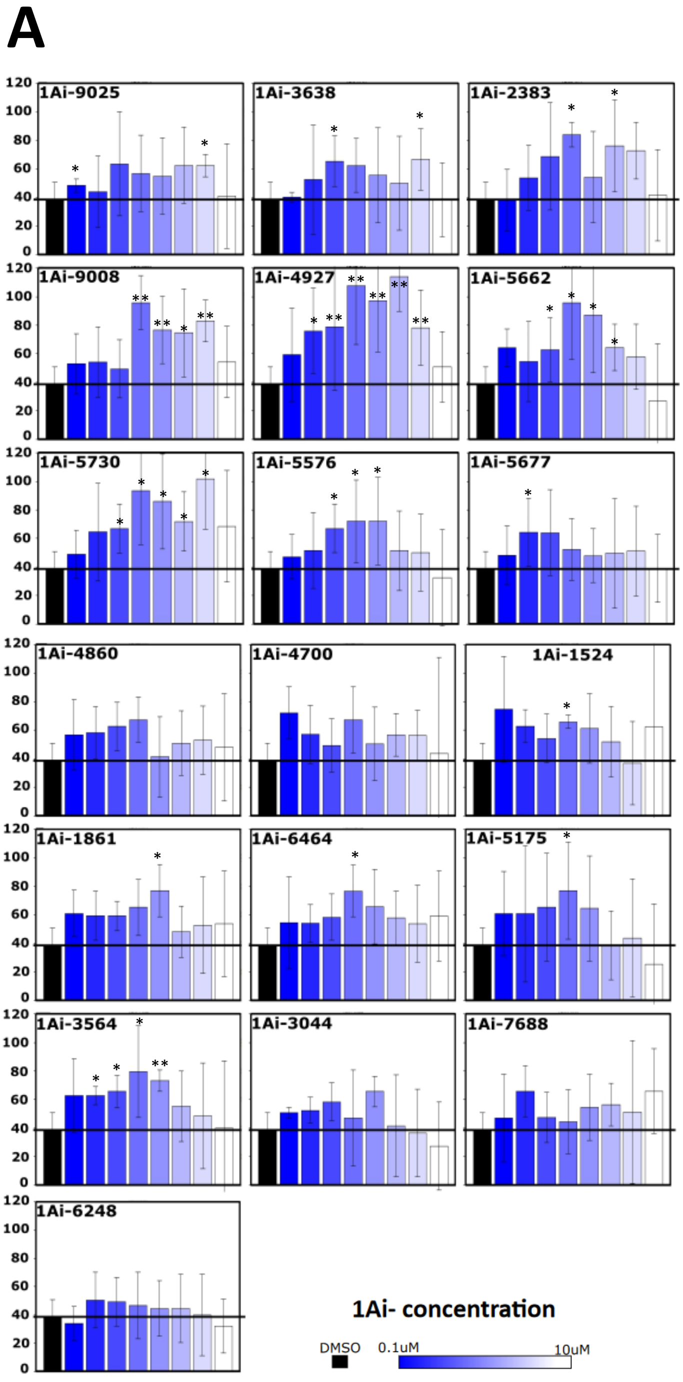

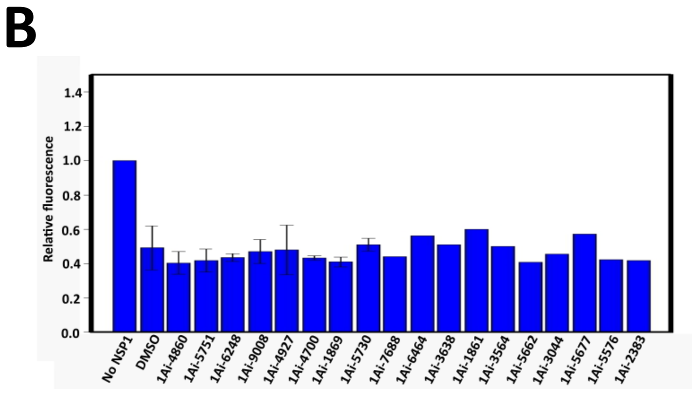

Appendix Figure S7: (A) 1Ais increases cellular viability after Covid-19 infection. Vero E6 cells were incubated with increasing concentrations of 1Ais for 1 hour and infected by the COVID-19 virus. After 72 hours cellular viability was measured. 100% represent uninfected cells; Error bars represent SD, n=3 independent biological replicates, $^*p<0.05$, $^{**}P<0.01$, student t-test. (B) 1Ais do not relieve a NSP1 inhibition of translation. HEK293 Cells were transfected with NSP1 and a GFP reporter which is sensitive to NSP1 expression. Cells were incubated overnight with 1Ais and GFP fluorescence intensity was measured; n=6 independent biological replicates for DMSO and No-NSP1, n≥1 for 1Ais treatments.

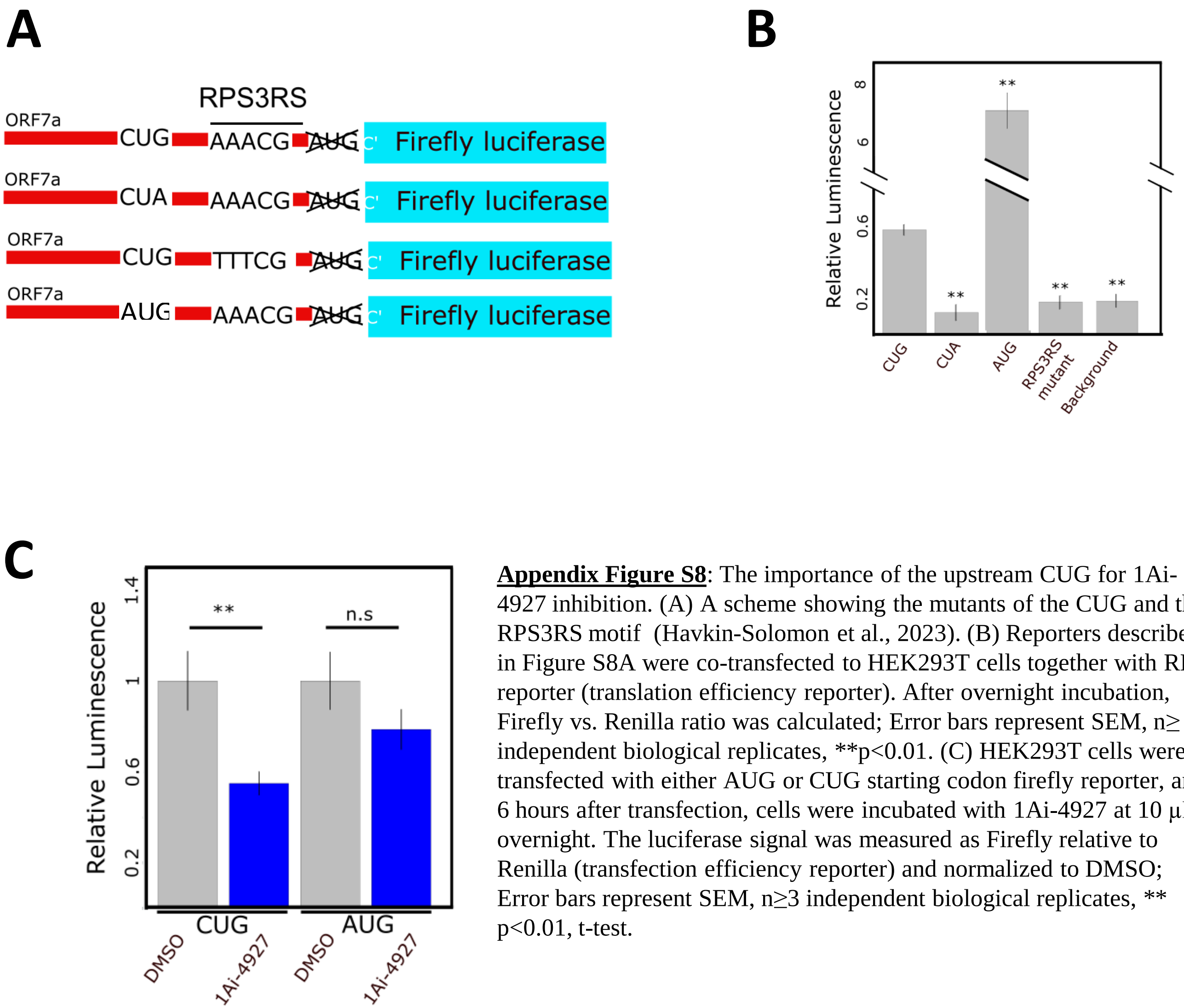

<u>Appendix Figure S8</u>: The importance of the upstream CUG for 1Ai-4927 inhibition. (A) A scheme showing the mutants of the CUG and the RPS3RS motif (Havkin-Solomon et al., 2023). (B) Reporters described in Figure S8A were co-transfected to HEK293T cells together with RL reporter (translation efficiency reporter). After overnight incubation, Firefly vs. Renilla ratio was calculated; Error bars represent SEM, n≥ 3 independent biological replicates, **p<0.01. (C) HEK293T cells were transfected with either AUG or CUG starting codon firefly reporter, and 6 hours after transfection, cells were incubated with 1Ai-4927 at 10 µM overnight. The luciferase signal was measured as Firefly relative to Renilla (transfection efficiency reporter) and normalized to DMSO; Error bars represent SEM, n≥3 independent biological replicates, ** p<0.01, t-test.

Appendix Table S1 – 1Ais structures and IC50 (uM) from the split RL screen

1Ai	IC50	Structure
1Ai-4860	23	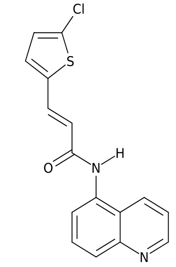
1Ai-5576	24	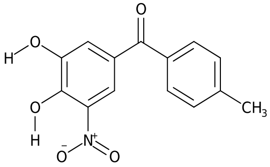
1Ai-3564	15	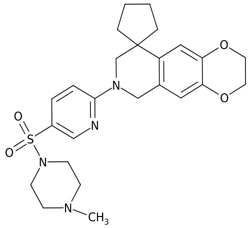
1Ai-9162	16	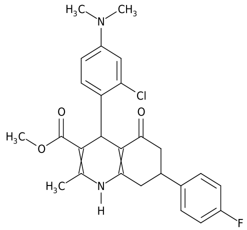

1Ai	IC50	Structure
1Ai-3638	23	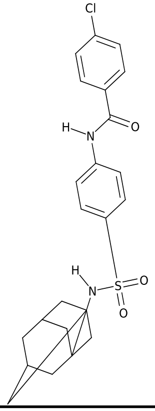
1Ai-9008	27	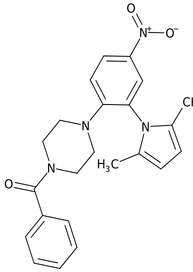
1Ai-5677	15	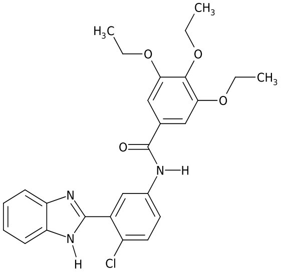
1Ai-1861	14	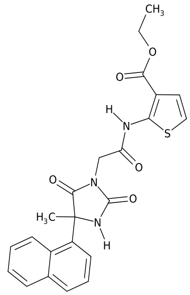

1Ai	IC50	Structure
1Ai-4700	11	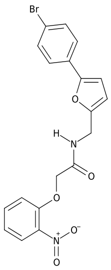
1Ai-1524	30	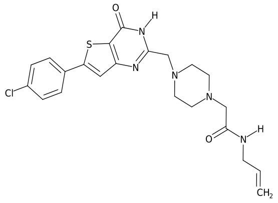
1Ai-0403	17	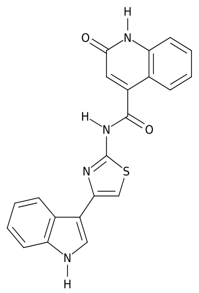
1Ai-3044	15	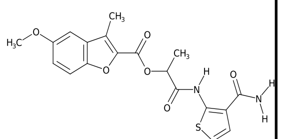

1Ai	IC50	Structure
1Ai-5751	18	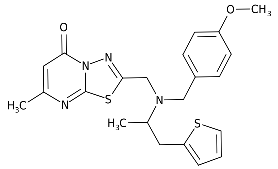
1Ai-7688	20	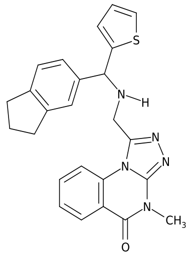
1Ai-6476	15	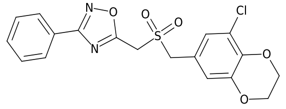
1Ai-2383	23	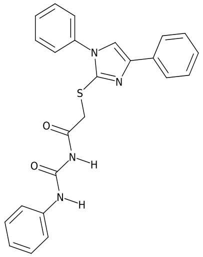

1Ai	IC50	Structure
1Ai-4927	14	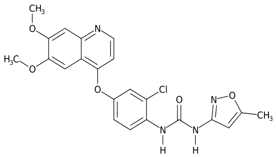
1Ai-5730	30	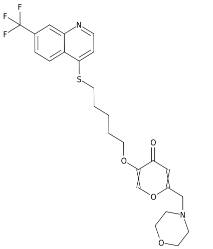
1Ai-5662	25	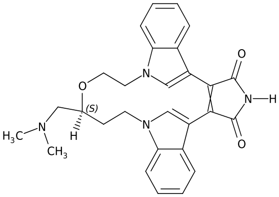
1Ai-8214	17	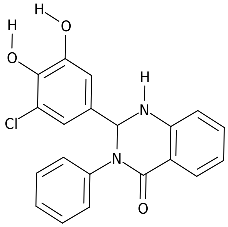

1Ai	IC50	Structure
1Ai-0050	26	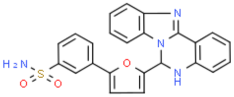
1Ai-6464	13	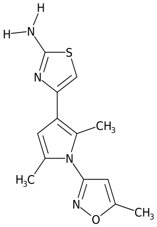
1Ai-5175	16	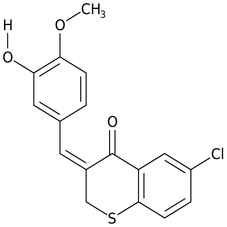
1Ai-6248	28	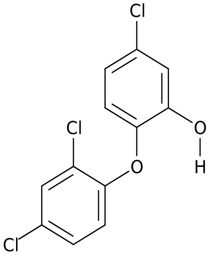

1Ai	IC50	Structure
1Ai-9025	17	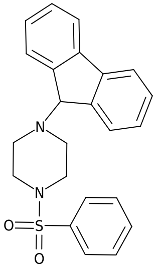
1Ai-2136	29	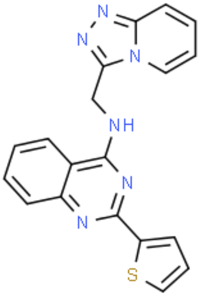
1Ai-0153	27	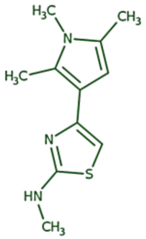
1Ai-6586	24	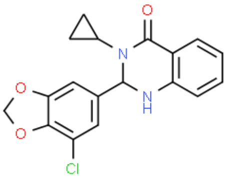

1Ai	IC50	Structure
1Ai-1869	18	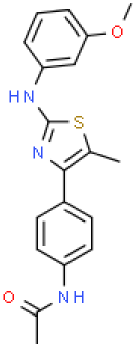

Appendix Table S2- Cloning primers

Experiment	Name	Sequence
In vitro expression of eIF1A and RPS10 for drug screen. Cloning eIF1A and RPS10 with split Renilla from mammalian vector to PRSFduet vector for bacterial expression, preparation of empty split Renilla and removing His-tag.	eIF1aΔC_F	CATCACCATCATCACCACAGCCAGGATCCGatgcccaagaataaaggtaaag
	eIF1aΔC_R	CCCGTTAGTAAAAGGTGGTTAAAAGCTTGCGGCCGCATAATGCTTAAGTCGA
	RPS10ΔN_F	ATTAGTTAAGTATAAGAAGGAGATATACATATGTTGATGCCTAAGAAGAAC
	RPS10ΔN_R	CGCAGCAGCGGTTTCTTTACCAGACTCGAGTTATTGTTCATTTTTGAGAAC
	EmptyΔC_F	CATCACCATCATCACCACAGCCAGGATCCGATGACTTCGAAAGTTTATGATC
	EmptyΔC_R	TCGACTTAAGCATTATGCGGCCGCAAGCTTTTAACCACCTTTTACTAACGGG
	EmptyΔN_F	ATTAGTTAAGTATAAGAAGGAGATATACATATGAAACCTGACGTTGTACAAATTG
	EmptyΔN_R	GTTCTCAAAAATGAACAATAACTCGAGTCTGGTAAAGAAACCGCTGCTGCG
	Remove_his_F	CTTTAATAAGGAGATATACCATGcccaagaataaaggtaaagg
	Remove_his_R	TCGACTTAAGCATTATGCGGCCGCAAGCTTTTAACCACCTTTTACTAACGGG

Experiment	Name	Sequence
Bacterial expression for protein purification.<br>eIF1A and RPS10 sequences taken from PRSFduet plasmid and cloned into bdsumo pet28c.	1A_tosumo_F	ATCGACGCAATGCTTCATCAGACTGGTGGCATGCCCAAGAATAAAGGTAAAG
	1A_tosumo_R	GATCTCAGTGGTGGTGGTGGTGGTGCTCGAGTTAGATGTCATCAATATCTTCAT
	RSP10_F	TTTAACTTTAAGAAGGAGATATACCATGTTGATGCCTAAGAAGAACC
	RSP10_R	CGGCACCAGTCAGCTGCTGTGATGATGATGATGATGCTGAGGTGGCTGACCACGT
Truncation of CTT or NTT of eIF1A based on bdSumo-1A in pet28c from previous cloning.	Remove_NTT_F	GATGGTCAGGAGTATGCTC
	Remove_NTT_R	GCCACCAGTCTGATGAAG
	Remove_CTT_F	TAAAAGCTTGCGGCCGCA
	Remove_CTT_R	TTTAGCATGCTCTGGAAGCTC

Experiment	Name	Sequence
Cloning ORF7a before Firefly/Renilla constructs for 1Ai-4927 and COV19 experiments. COV19 orf7a 5’UTR was taken from SARS-COV2 gmRNA 5’UTR and cloned before a standard firefly.	ORF7a_To_FF_F	GATCTGTTCTCTAAACGAACatgcgaagacgccaaaaacata
	ORF7a_To_FF_R	CGAGCGATCGCCTAGAATTACACGGCGATCTTTCC
Cloning uORF -46 and -95 nt before main ORF starting with the reference 5’UTR (Figure 4J).<br>To create the -95 we used the -46 and added 49 nt sequence form GFP without any start or stop codons (GCAACATCCTGGGGCACAAGCTGGAGTACAACTACAACAGCCACAACGT)	REF_-46_F	TAGGCTTTTGCAAAAAGCATGATTCTTCTGACACAAC
	REF_-46_R	GCCTACTGCAGCTTAAG
	Lift_GFP_F	CACAACAGTCTCGAACT**TAA**GCAACATCCTGGGGCACA
	Lift_GFP_R	CCATGGTGGCCTACTGCAGCACGTTGTGGCTGTTGTAGTT
Removing stop codon of the aforementioned -46 uORF plasmid.	REF_-46_leaky_F	GACACAACAGTCTCGAACTATAAGCTGCAGTAGGCCAC
	REF_-46_leaky_R	CTATAAGCTGCAGTAGGC
Changing IRF7 to Kozak and changing position +5 to C. Next, adding barcodes after the stop codon of the CDS.	IRF7_kozak_F	GACGGAGCGGATC**A**AAATG**GA**AGACGCCA
	IRF7_c5_F	CGGAGCGGATC**C**AAATG**GC**AGACGCCAAAAACAT
	IRF7_kozak_C5_F	CGGAGCGGATC**A**AAATG**GC**AGACGCCAAAAACAT
	IRF7_all-mut_R	CCATCCTCTAGAGGATAG
	IRF7_b1	TAACTGTATTCAGCGATGACAGCTGCAGCTATTGTAATCCTCCG
	IRF7_kozak_b2	TAACTGTATTCAGCGATGACTGAACGCGCTAGCTATTGTAATCCTCCG
	IRF7_c5_b3	TAACTGTATTCAGCGATGACTCGGATCCAGAGCTATTGTAATCCTCCG
	IRF7_kozak_C5_b4	TAACTGTATTCAGCGATGACCCATAACGGTAGCTATTGTAATCCTCCG
	Barcodes_R	CCCTGAACCTGAAACATAA

Experiment	Name	Sequence
Mutating ORF7a of SARS-COV2 at the CUG start codon and regulatory element	CUG_to_CUA	CTTTCGATCTCTTGTAGATCTATTCTCT AAACGAACATGGC
	CUG_to_AUG	CTTTCGATCTCTTGTAGATATGTTCTCT AAACGAACATGGC
	AAACG_to_TTTCG	CGATCTCTTGTAGATCTGTTCTTTTCG AACATGGCAAGACGCCA
	Reverse_mut	CCCTGAACCTGAAACATAA
qPCR primers for firefly luciferase reporter	FF_Forward	AACATTTCGCAGCCTACCGTAGTG
	FF_Reverse	AAACCGGGAGGTAGATGAGATGTG
